# Identification of a Novel Sequence Motif Recognized by the Ankyrin Repeat Domain of zDHHC17/13 *S*-Acyltransferases*

**DOI:** 10.1074/jbc.M115.657668

**Published:** 2015-07-21

**Authors:** Kimon Lemonidis, Maria C. Sanchez-Perez, Luke H. Chamberlain

**Affiliations:** From the Strathclyde Institute of Pharmacy and Biomedical Sciences, Univesity of Strathclyde, Glasgow G4 0RE, United Kingdom

**Keywords:** Golgi, Huntington disease, membrane enzyme, protein palmitoylation, substrate specificity, S-acylation, ankyrin repeat domain, zDHHC13, zDHHC17

## Abstract

*S*-Acylation is a major post-translational modification affecting several cellular processes. It is particularly important for neuronal functions. This modification is catalyzed by a family of transmembrane *S*-acyltransferases that contain a conserved zinc finger DHHC (zDHHC) domain. Typically, eukaryote genomes encode for 7–24 distinct zDHHC enzymes, with two members also harboring an ankyrin repeat (AR) domain at their cytosolic N termini. The AR domain of zDHHC enzymes is predicted to engage in numerous interactions and facilitates both substrate recruitment and *S*-acylation-independent functions; however, the sequence/structural features recognized by this module remain unknown. The two mammalian AR-containing *S*-acyltransferases are the Golgi-localized zDHHC17 and zDHHC13, also known as Huntingtin-interacting proteins 14 and 14-like, respectively; they are highly expressed in brain, and their loss in mice leads to neuropathological deficits that are reminiscent of Huntington's disease. Here, we report that zDHHC17 and zDHHC13 recognize, via their AR domain, evolutionary conserved and closely related sequences of a [VIAP][VIT]*XX*QP consensus in SNAP25, SNAP23, cysteine string protein, Huntingtin, cytoplasmic linker protein 3, and microtubule-associated protein 6. This novel AR-binding sequence motif is found in regions predicted to be unstructured and is present in a number of zDHHC17 substrates and zDHHC17/13-interacting *S*-acylated proteins. This is the first study to identify a motif recognized by AR-containing zDHHCs.

## Teaser

**Background:** *S*-Acylation, a protein lipidation process that is essential for neuronal functions, is catalyzed by zDHHC *S*-acyltransferases.

**Results:** The ankyrin repeat (AR) domains of zDHHC17 and zDHHC13 recognize a novel unstructured peptide sequence in several unrelated proteins.

**Conclusion:** Several proteins have independently acquired similar short peptide sequences for zDHHC17/13 binding.

**Significance:** This is the first study to identify a motif recognized by AR-containing *S*-acyltransferases.

## Introduction

Protein *S*-acylation (also known as palmitoylation) is a prominent post-translational modification in eukaryotes involved in the regulation of protein trafficking, localization, stability, and function; this process is catalyzed by a family of transmembrane *S*-acyltransferases (zDHHCs)^2^ that share a conserved catalytic zinc finger DHHC (Asp-His-His-Cys)-containing domain (1–3). Based on this domain, several zDHHC enzymes (ranging from 7 to 24 per organism) have been identified and characterized in various animal, parasite, and fungal species. Mammalian (4), fly (5), nematode worm (6), apicomplexan parasite (7), and yeast (8) species have been shown to contain two zDHHC enzymes that also harbor an ankyrin repeat (AR) domain at their cytosolic N termini. AR domains on zDHHCs can act as substrate-recruiting modules for *S*-acylation (see below) but may also participate in *S*-acylation-independent functions, such as formation of JNK-MKK7 complex for JNK activation (9, 10) and suppression of heterotrimeric G-protein signaling by sequestration of Gβγ dimer (11). In mammals, these two AR-containing zDHHCs are known as zDHHC17 and zDHHC13, or Huntingtin-interacting protein 14 (HIP14) and 14-like (HIP14L), respectively. These are both Golgi-localized neuronal *S*-acyltransferases with a seven-AR domain, and their loss in mice results in numerous synaptic, memory, locomotion and behavior deficits, reminiscent of Huntington's disease (12–16). zDHHC17 functions are thought to be maintained through vertebrate evolution because of the very high sequence conservation among its distal vertebrate orthologues (17). To date, zDHHC17 has been shown to recruit via its seven-AR domain, and subsequently *S*-acylate, four neuronal proteins: Huntingtin (HTT) (18, 19), JNK3α2 (9, 20), CSPα, and SNAP25b (21). zDHHC13 has been reported to also bind the above substrates (9, 21, 22), but, with the exception of HTT (18, 19), these interactions were not shown to result in significant *S*-acylation of these proteins.

AR domains are well known protein-protein interaction modules (23), and indeed a recent yeast two-hybrid screen identified numerous zDHHC17 AR-interacting proteins (24); however, the structural/sequence elements required for binding to the AR domain of zDHHC enzymes are currently not known. Here, we report that zDHHC17 and zDHHC13 recognize a novel sequence motif in a number of proteins previously found to interact with zDHHC17.

## Experimental Procedures

### 

#### 

##### Chemicals

Ni^2+^-NTA-agarose was purchased from Qiagen, glutathione-Sepharose 4B was from GE Healthcare, yeast nitrogen base and CSM-Ade,-His,-Trp,-Leu,-Ura,-Met drop-out mix was from MP Biomedicals, (NH_4_)_2_SO_4_ and BSA standards were from Fisher Scientific, and agar was from Oxoid (Basingstoke, UK). Unless otherwise stated, all other chemicals were purchased from Sigma.

##### Antibodies

Rabbit VP16 (ab4808) was from Abcam (Cambridge, UK), rat HA was from Roche, mouse GFP (JL8) was from Clontech, and mouse FLAG M2 was from Sigma. Secondary IRDye mouse and rabbit antibodies were from LI-COR (Cambridge, UK), whereas DyLight rat secondary antibody was from Fisher Scientific.

##### Cloning and Mutagenesis

With the exception of GST fusion proteins (described below), all cDNAs were cloned by Gateway Technology (21), using manufacturer's kits and instructions (Life Technologies, Inc.). PCR and site-directed mutagenesis reactions were performed using a KOD hot start polymerase kit according to the manufacturer's guidelines (Merck Millipore, Watford, UK). Primers for introducing Gateway compatible adapters by PCR (attB-PCR) were purchased from Life Technologies, Inc., whereas primers for site-directed mutagenesis were purchased from Sigma. Plasmids were sequenced by GATC (Constance, Germany). Original cDNAs used for cloning were as follows: murine zDHHC17 (DHHC17), zDHHC13 (DHHC22), and zDHHC3 (DHHC3) clones in an HA-pEF-BOS vector were kindly provided by Prof. Masaki Fukata (4), whereas rat SNAP25b, bovine CSPα, and mouse SNAP23 cDNA were as previously described (25–27); human MAP6 (full-length N-STOP isoform) cDNA was recovered from a human embryonic brain cDNA library, whereas E-STOP cDNA was generated by site-directed mutagenesis introducing a STOP codon at position Ser^440^ of N-STOP cDNA; N-terminal human HTT (corresponding to 1–550 amino acids with 23 Q repeats; UniProt ID: P42858) and human codon-optimized CLIP3 cDNAs were synthesized by Life Technologies, Inc. All site-directed mutagenesis reactions occurred in entry clones and were confirmed by sequencing. The GST-17NAnk construct has been described before (21). The GVVASQPARV sequence of SNAP25b (SNAP25b_111–120_) was appended to the C terminus of GST by introduction of appropriate codons on the pGEX-KG polycloning site using site-directed mutagenesis. Full-length rat SNAP25b was subcloned into the pGEX-KG polycloning site (for expression of GST-SNAP25b_FL_ protein), by introduction of HindIII and SalI sites by PCR, followed by restriction digestion of the SNAP25b PCR product and pGEX-KG vector and subsequent ligation of the two fragments. Plasmids pGST-IRAP_78–109_ (28) and pFLAG-TNKS-2 (29) were kindly provided by Nai-Wen Chi.

##### Protein Purification

BL21(DE3)pLysS bacterial cells (Life Technologies, Inc.) were transformed with plasmids encoding for GST, GST-17Ank, GST-SNAP25b_111–120_, GST-SNAP25b_FL_, GST-IRAP_78–109_. His_6_-CSPα, His_6_-SNAP25b_93–206_,and His_6_-AR_D17_ (residues 54–288); transformed cells were selected with appropriate antibiotic and expression of proteins was induced by isopropyl β-d-thiogalactopyranoside. Cells expressing the corresponding proteins were collected by centrifugation, resuspended in binding buffer (PBS for GST-glutathione binding, and 20 mm Tris-HCl, pH 8, 150 mm NaCl, 10 mm imidazole for six-histidine Ni^2+^-NTA binding), and subsequently lysed by a 30-min incubation on ice with 1 mg/ml lysozyme followed by sonication. After clarification by centrifugation (20,000 × *g*, for 40 min at 4 °C), bacterial lysates were loaded to either glutathione resin (GST and GST-tagged proteins/peptides) or Ni^2+^-NTA resin (His_6_-CSPα and His_6_-SNAP25b_93–206_). GST and GST-17NAnk were eluted in 50 mm Tris pH 8, 10 mm Glutathione, after extensive washes of the glutathione-Sepharose beads with PBS. His_6_-CSPα, His_6_-SNAP25b_93–206_ and His_6_-AR_D17_ were eluted in 20 mm Tris-HCl pH 8, 150 mm NaCl, 500 mm Imidazole, after extensive washing of Ni^2+^-NTA-agarose beads with 20 mm Tris-HCl, pH 8, 300 mm NaCl, 50 mm imidazole. Eluted proteins were dialyzed overnight against 5 liters of PBS or Tris buffer (20 mm Tris-HCl pH 7.6, 150 mm NaCl), and their concentration was estimated from the intensity of their corresponding Coomassie-stained bands (following SDS-PAGE), as compared with the standard curve obtained by BSA standards that were run in parallel.

##### Identification of MAP6 as a Potential AR_D17_-binding Protein

From a previous GST-17NAnk pulldown of rat brain proteins (21), bound proteins were loaded for SDS-PAGE and were visualized by Coomassie staining. Gel slices containing protein bands that were missing from control GST pulldowns were sent for mass spectrometry identification (University of Glasgow). Among the various peptides identified from a slice of approximate 50 kDa, there were four high confidence peptides corresponding to rat MAP6, with a probability Based Mowse score of 186.

##### Split Ubiquitin System (SUS)

Yeast matings expressing both zDHHC-Cub-PLV baits and NubG-2HA-tagged preys were verified in synthetic defined medium (0.17% (w/v) yeast nitrogen base without (NH_4_)_2_SO_4_, 0.5% (w/v) (NH_4_)_2_SO_4_, 2% (w/v) glucose, 0.15% (w/v) CSM-Ade,-His,-Trp,-Leu,-Ura,-Met drop-out mix, and 2% (w/v) agar, pH 6), supplemented with adenine sulfate (Ade; 0.002% w/v) and histidine (0.002% w/v). 5 μl of matings at corresponding *A*_600_ were dropped on synthetic defined medium to assess interactions (resulting in PLV-dependent transcriptional activation of auxotrophy genes), and on synthetic defined medium supplemented with Ade and His to verify equal optical density among matings. The ability of each bait (zDHHC-Cub-PLV) to promote a transcription response with Nub in the absence of prey (NubG-2HA-tagged protein) was assessed with negative control, NubG-2HA (HA-tagged Nub having a I13G mutation) and positive control, NubI (nonmutated/wild-type Nub). However, because the association with Nub could vary among different baits (because of differential bait expression and/or possible structural constraints), when interactions of different baits was compared, the corresponding matings were grown for the appropriate number of days to ensure equal growth for all baits expressed with NubI (21). Expression of zDHHC-Cub-PLV (baits) and NubG-2HA-tagged proteins (preys) in euploid yeast cells, was assessed by Western blotting using VP16 and HA antibodies, respectively. Unless otherwise stated, proteins were run on 12% SDS-polyacrylamide gels, prior to transfer to nitrocellulose (Bio-Rad) and Western blotting. Yeast lysis for Western blotting analysis has been described previously (30). The principle of the mating-based SUS and its application for the assessment zDHHC substrate specificity can be found elsewhere (21), whereas a more comprehensive procedure of this technique has been published before (30).

##### Pulldown Assays

For CSPα-SNAP25b competition for binding to the AR of zDHHC17, 10 μl of glutathione-Sepharose resin was incubated with 20 μg of GST-17NAnk (or GST) for 4 h at 4 °C, and then with 20 μg of His_6_-CSPα or His_6_-SNAP25b_93–206_ (and competing amount of His_6_-SNAP25b_93–206_ or His_6_-CSPα) overnight at 4 °C. After extensive washing with PBS, bound proteins were eluted after boiling the glutathione-Sepharose beads in 100 μl of Laemmli sample buffer. 7.5% of total input and 15% of bound fractions were loaded on 16% SDS-polyacrylamide gels; following SDS-PAGE, gels were stained with Bio-Safe Coomassie (Bio-Rad) and visualized using an Odyssey LI-COR infrared imager.

For GST pulldowns of AR-containing proteins, HEK293T cells expressing the corresponding HA-tagged or FLAG-tagged proteins in 24-well plates were lysed by addition of 200 μl of Tris buffer (20 mm Tris-HCl, pH 7.6, 150 mm NaCl) supplemented with 0.5% (see Fig. 5*B*) or 1% (see Fig. 5*A*) Triton X-100; after the addition of protease inhibitors and clarification by centrifugation (10,000 × *g* for 10 min at 4 °C), 180 μl of the corresponding lysate was then incubated overnight at 4 °C with 80 μl of glutathione-Sepharose resin and 125 μg of corresponding GST fusion protein, each diluted in Tris buffer. After extensive washes with Tris buffer (see Fig. 5*B*) or Tris buffer supplemented with 0.5% Triton X-100 (see Fig. 5*A*), bound proteins were eluted by boiling in Laemmli sample buffer. Following SDS-PAGE on 12% gels and transfer to nitrocellulose, bound GST proteins were detected by Ponceau S staining and HA/FLAG-tagged AR-containing proteins by Western blotting using an HA or a FLAG antibody.

For His_6_-AR_D17_ pulldowns, HEK293T cells expressing the corresponding EGFP-tagged proteins in 6-well plates were lysed by the addition of 600 μl of lysis buffer (20 mm Tris-HCl, pH 8, 150 mm NaCl, 1% Triton X-100, 20 mm imidazole); 220 μl of the corresponding lysate was then incubated with 25 μl of Ni^2+^-NTA-agarose resin and 30 μl (75 μg) of His_6_-AR_D17_ (or equivalent volume of PBS control) for 2 h at 4 °C. After extensive washing with washing buffer (20 mm Tris-HCl, pH 8, 300 mm NaCl, 1% Triton, 60 mm imidazole), bound proteins were eluted by boiling in 60 μl of Laemmli sample buffer. 4% of total input and 20% of bound fractions were loaded on 12% SDS-polyacrylamide gels, and following SDS-PAGE and transfer to nitrocellulose, bound His_6_-AR_D17_ were detected by Ponceau S staining and EGFP-tagged proteins by Western blotting using a GFP antibody.

##### Secondary Structure and Disorder Prediction

To assess whether the Ψβ*XX*QP motif of proteins is part of any structural fold or lies in unstructured and possibly disordered regions, we first used DISOPRED3 (within the PSIPRED server). This tool takes into account evolutionary conserved disordered regions of missing residues in x-ray structures and predicts both protein secondary structure and disorder with a precision (75%) that is the highest among current disorder prediction platforms (31, 32). For proteins whose Ψβ*XX*QP motif was predicted to lie within regions lacking a secondary structure (coils), but not being disordered, we also utilized the PrDOS tool (Protein DisOrder Prediction); PrDOS uses a similar approach to DISOPRED3 but without giving any information about secondary structure; although its precision (70%) is a bit lower than DISOPRED3, it has a slightly improved accuracy, allowing for the prediction of additional disordered regions that could have been missed by DISOPRED3 as false negatives (31, 33). DISOPRED3 and PrDOS have been evaluated as the two most reliable disorder prediction tools, among the 28 prediction groups tested, by the recent Critical Assessment of techniques for protein Structure Prediction, CASP10 (31).

##### Identification of zDHHC17-interacting Proteins Having a ΨβXXQP Motif

To identify zDHHC17/13-interacting proteins that are highly likely to interact with these zDHHCs via a Ψβ*XX*QP motif, we searched for this motif in proteins that are either confirmed to interact with zDHHC17/13 or have higher probability to be interacting with these; we thus focused on the recently identified zDHHC17 interactors from a yeast two-hybrid screen, which are either *S*-acylated or confirmed to interact with zDHHC17 by another independent assay and the previously published zDHHC17/13 interactors/substrates (24); in addition, we also focused on closely related homologues of these proteins, proteins whose *S*-acylation is decreased in zDHHC17-deficient mice (34), as well as the sole published zDHHC13-only interactor (matrix metalloproteinase 14) (35). We initially found 17 zDHHC17-interacting proteins to have such motifs; however, after filtering these proteins for their ability to bind zDHHC17 under physiological conditions (evidence for Golgi or plasma membrane localization, *S*-acylation, interaction with zDHHC13, or homology to any protein with these features), this number dropped to 15. When we additionally tested whether the Ψβ*XX*QP motif of each protein is expected to lie within disordered regions (DISOPRED3 and PrDOS disorder prediction), this number dropped further to 14. Along with the 6 zDHHC17/13-interacting proteins identified in this study, these additional 14 proteins were also considered highly probable to interact with zDHHC17/13 via a linear Ψβ*XX*QP-containing sequence (see Table 1).

## Results

### 

#### 

##### Regions of Sequence Homology within CSPα and SNAP25b Are Involved in Binding to the AR Domain of zDHHC17/13

**FIGURE 1.**
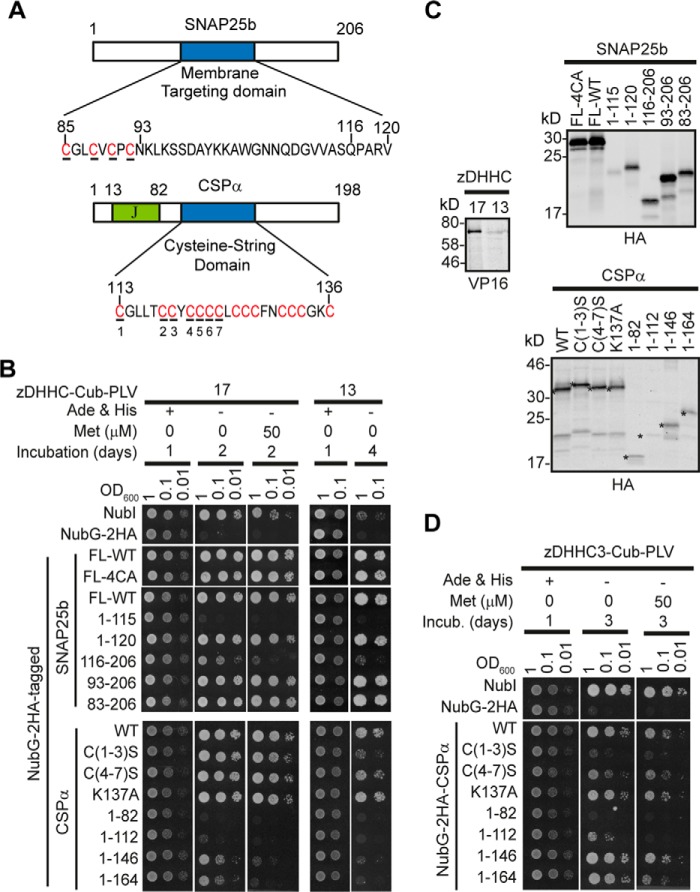
**Identification of regions of SNAP25b and CSPα required for interaction with zDHHC17 and zDHHC13.** *A*, schematic diagram of rat SNAP25b and bovine CSPα domains, with *S*-acylated cysteines shown in *red*; cysteines mutated [4CA in SNAP25b, and C(1–3)S and C(4–7)S in CSPα] are *underlined. B*, assessment of SNAP25b and CSPα mutant interactions with zDHHC17/13 using the SUS; growth conditions are shown on the *top. C*, euploid yeast lysate proteins were resolved in 12% gels (or 4–20% for CSPα constructs), and proteins were visualized following Western blotting with HA and VP16 antibodies. *Asterisks* indicate expected products for CSP mutants. *D*, assessment of zDHHC3 interaction with CSPα mutants in SUS, as in *B*.

We have previously shown that SNAP25b and CSPα, although being *S*-acylated by many Golgi zDHHC enzymes (26, 27), are specifically recruited by the AR domains of zDHHC17 and zDHHC13 (21). To identify the regions of SNAP25b and CSPα that bind to the AR domain of zDHHC17/13, we created a series of truncated and point mutants of these proteins and assessed their binding to zDHHC17 and zDHHC13 (Fig. 1, *A* and *B*), using the mating-based SUS in yeast (30), which we have previously evaluated for assessment of zDHHC substrate specificity (21). As expected, deletions of SNAP25b that left the 85–120 minimal membrane targeting domain intact (*i.e.* 1–120 and 83–206 mutants) did not affect its interaction with either zDHHC17 or zDHHC13; SNAP25b interactions were also independent of its *S*-acylated cysteine-rich region (present within the minimal membrane targeting domain), because neither alanine substitution (FL-4CA mutant) nor deletion of the N terminus encompassing these cysteines (93–206 mutant) impaired its binding to zDHHC17/13; however, C- or N-terminal truncations from or before residue Gln^116^ (*i.e.* 1–115 and 116–206) rendered SNAP25b unable to bind either zDHHC17 or zDHHC13, which suggests that the region around Gln^116^ is involved in recognition by zDHHC17/13. These results are consistent with the role of the 93–120 region of SNAP25b and particularly of amino acids Val^113^, Gln^116^, Pro^117^, and Val^119^ in membrane targeting and *S*-acylation by zDHHC17 (36, 37). CSPα mutations C(1–3)S, C(4–7)S, and K137A, previously found to affect its initial membrane binding and/or *S*-acylation (25), had minimal or no effect on its interaction with zDHHC17/13; in contrast, all C-terminal truncations tested (*i.e.* 1–82, 1–112, 1–146, and 1–164 mutants) resulted in a marked loss of interaction with both zDHHC17 and zDHHC13, suggesting that amino acids in the region 165–198 of CSPα are involved in interaction with these enzymes. This region of CSPα is specifically required for binding to zDHHC17/13, because the weak interaction of zDHHC3 with CSPα was not affected by C-terminal truncations downstream of its cysteine string domain but was instead perturbed by serine substitution of the first seven cysteine residues within this domain (Fig. 1*D*).

**FIGURE 2.**
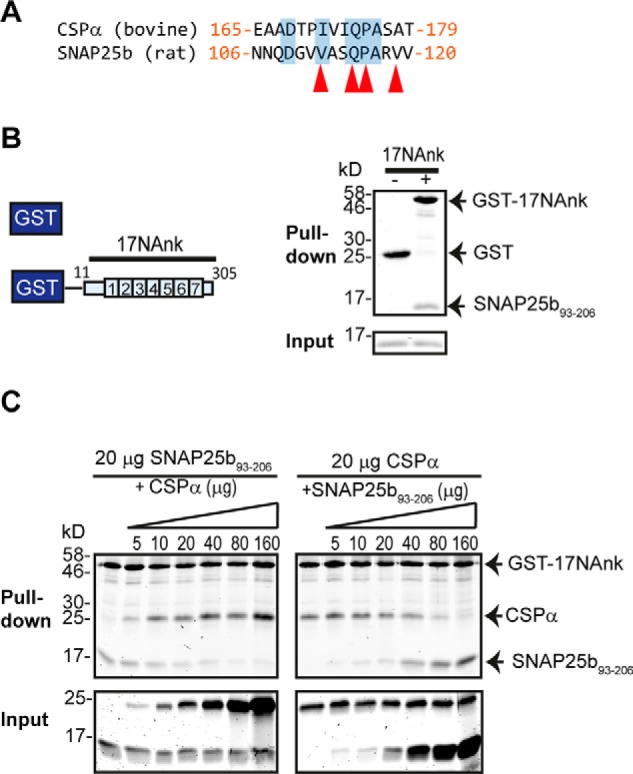
**CSPα and SNAP25b occupy the same binding site on zDHHC17.** *A*, sequence alignment of CSPα (165–179) with SNAP25b (106–120). Similar/identical amino acids are highlighted in *blue shading*, and those found to be involved in membrane targeting of SNAP25b are indicated with *red arrows. B*, pulldown assay. Purified His-tagged SNAP25_93–206_ can bind to GST-tagged N-terminal (1–305) zDHHC17 (17NAnk) but not to GST alone. Proteins were resolved in 16% gels, stained with Coomassie Blue, and visualized using a LI-COR infrared imager. *C*, competition between His-tagged SNAP25b_93–206_ and His-tagged CSPα for binding to limiting amounts of GST-17NAnk (20 μg). Proteins were resolved and visualized as in *B*.

Inspection of the amino acid regions in CSPα (residues 165–198) and SNAP25b (residues 93–120) required for zDHHC17/13 interaction revealed a short region of sequence similarity between these proteins (Fig. 2*A*), including amino acids (Val^113^, Gln^116^, and Pro^117^) previously shown to be important for membrane targeting of SNAP25 and its *S*-acylation by zDHHC17 (37). This implies that a [VI]*XX*QP motif within SNAP25b and CSPα may be involved in zDHHC17/13 binding. If the AR of zDHHC17/13 recognizes the same motif on both SNAP25b and CSPα, then these proteins should occupy the same binding site on zDHHC17/13. To assess this possibility, we performed a competition assay between His-tagged SNAP25b_93–206_ and CSPα, for binding to limiting concentrations of a GST-tagged AR-containing cytosolic region of zDHHC17 (GST-17NAnk). As shown before for full-length CSPα and SNAP25b (21) and here for truncated SNAP25, SNAP25b_93–206_ (Fig. 2*B*), these proteins bind specifically to GST-17NAnk and not to GST-bound glutathione beads. Increasing amounts of either CSPα or SNAP25b_93–206_ out-competed each other for binding to GST-17NAnk (Fig. 2*C*), strongly suggesting that a similar motif in CSPα and SNAP25b is recognized by a specific binding site in the zDHHC17/13-AR domain.

##### The AR Domain of zDHHC17/13 Recognizes Proteins Bearing ΨβXXQP-containing Unstructured Regions

**FIGURE 3.**
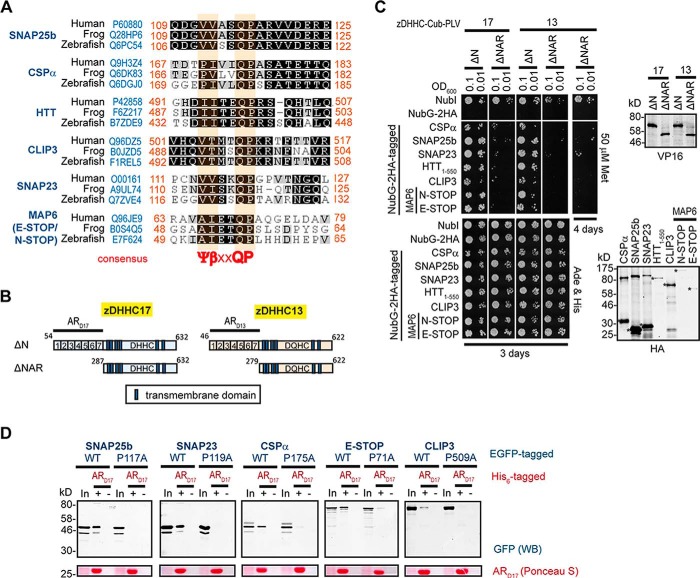
**The conserved proline within the Ψβ*XX*QP motif of AR_D17_-binding proteins SNAP25b, CSPα, MAP6, and CLIP3 is required for this interaction.** *A*, sequence alignment of human, frog, and zebrafish SNAP25b, CSPα, HTT, CLIP3, SNAP23, and MAP6 proteins reveals a homologous region of Ψβ*XX*QP consensus (Ψ indicates aliphatic Pro, Val, Ile, or Ala but not Leu; β indicates C-beta-branched Val, Ile, or The; *X* indicates any amino acid). Evolutionary conserved amino acids are highlighted (BLOSUM62 matrix: *black* indicates 100% similar, and *gray* indicates 60–80% similar). UniProt IDs and amino acid positions are shown. *B*, schematic diagram of zDHHC17/13 truncation mutants used in SUS, differing only in the existence of the AR domain. *C*, assessment of CSPα, SNAP25b, SNAP23, HTT_1–550_, CLIP3, N-STOP, and E-STOP interactions with zDHHC17/13 truncation mutants using the SUS. Corresponding Western blots with HA and VP16 antibodies are shown. *Asterisks* indicate expected products for different wild-type preys. *D*, pulldowns of wild-type and corresponding Pro to Ala mutants of EGFP-tagged SNAP25b, SNAP23, CSPα, E-STOP, and CLIP3, from HEK293T lysates, by Ni^2+^-NTA-bound His_6_-AR_D17_. Control pulldowns in the absence of His_6_-ARD17 were performed in parallel. 4% of total inputs (*In*) and 20% of pulled down fractions were run in 12% gels. Following transfer, blots were stained with Ponceau S solution and probed with GFP antibody.

**FIGURE 4.**
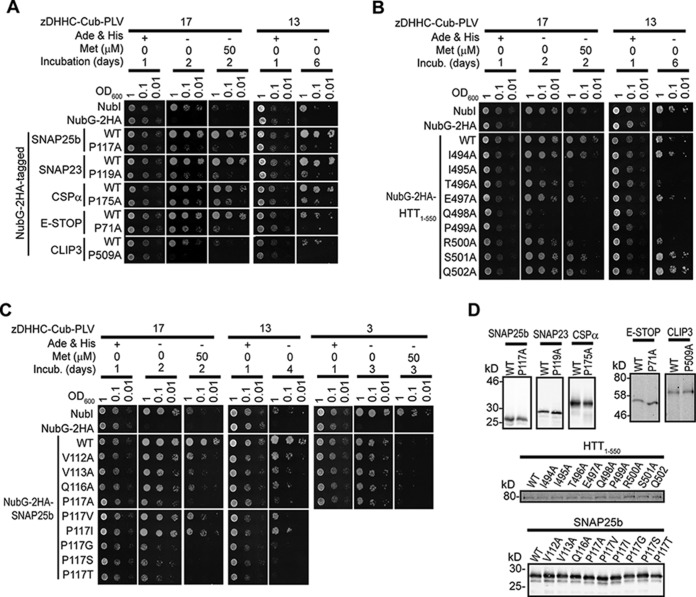
**Amino acid substitutions within the Ψβ*XX*QP motif of SNAP25b, CSPα, HTT, CLIP3, SNAP23, and MAP6 affect their interaction with zDHHC17 and zDHHC13.** Interactions of the above proteins (wild-type and point mutants) with zDHHC17/13 were assessed using the SUS. *A*, effect of mutating the conserved proline within the Ψβ*XX*QP motif of SNAP25b, SNAP23, CSPα, E-STOP, and CLIP3 on their interaction with zDHHC17 and zDHHC13. *B*, effect of individual alanine substitutions within a 494–502-amino acid region of HTT on zDHHC17/13 interaction. *C*, effect of amino acid substitutions within the Ψβ*XX*QP motif of SNAP25b on zDHHC17, zDHHC13, and zDHHC3 interaction. *D*, expression of NubG-HA-tagged proteins in euploid yeast, as assessed by Western blotting with an HA antibody. *Incub.*, incubation.

To assess whether the IVIQP and VASQP signatures in CSPα and SNAP25b, respectively, are part of a motif shared by other zDHHC17/13-AR binding proteins and whether this motif is indeed required for this binding, we looked for similar sequences in other proteins that are likely to interact with the AR domain of both zDHHC17 and zDHHC13. We initially focused on Huntingtin (HTT) and CLIP3 (cytoplasmic linker protein 3), because these two proteins have been found to interact with and be *S*-acylated by both zDHHC17 and zDHHC13 (19, 22, 38). Both HTT and CLIP3 have sequence signatures resembling those in CSPα and SNAP25b, which are located in regions previously proposed to bind these zDHHC enzymes: the IITEQP signature within the 1–548 amino acid region of HTT and a VTMTQP signature within the C-terminal membrane-binding domain of CLIP3. The ubiquitously expressed SNAP25 homologue SNAP23 also has a related VSKQP signature, which is required for its membrane targeting (27). Lastly, the neuronal Microtubule-Associated Protein 6 (MAP6; also known as STOP, stable tubule-only peptide), which we have identified as a zDHHC17-AR-binding protein (see experimental procedures), has a AIETQP signature proximal to its Golgi-targeting domain (39), present in both full-length N-STOP and shorter E-STOP isoforms (40). These six proteins collectively have sequences forming a Ψβ*XX*QP consensus (Ψ indicates aliphatic Val, Ile, Ala, or Pro; β indicates C-beta branched Val, Ile, or Thr; *X* indicates any amino acid), with individual amino acids in this region highly conserved among distal vertebrate species (Fig. 3*A*). These Ψβ*XX*QP-containing sequences do not seem to lie within any structural fold but are instead predicted to form intrinsically disordered coils (see “Experimental Procedures”). We found that all six proteins bearing Ψβ*XX*QP consensus sequences bind to the AR domain of zDHHC17/13, because they can all interact with AR-containing (full-length and truncated) zDHHC17 and zDHHC13 constructs in SUS (Fig. 3, *B* and *C*, and 4, *A* and *B*), but these interactions were greatly reduced upon removal of zDHHC17/13 AR domains (comparison between zDHHC17/13 ΔN and ΔNAR mutants) (Fig. 3, *B* and *C*). Additionally these proteins, with the exception of HTT_1–550_, could bind to the His-tagged-AR domain of zDHHC17, and this interaction was greatly impaired, or completely lost, when the conserved proline within the Ψβ*XX*QP consensus was mutated to alanine (Fig. 3*D*).

##### Importance of Individual Amino Acids within the ΨβXXQP Consensus for zDHHC17/13 Interaction

To examine the role of the conserved proline within the Ψβ*XX*QP consensus for both zDHHC17 and zDHHC13 binding and to also confirm the results of the His_6_-AR_D17_ pulldown assays (Fig. 3*D*), we compared zDHHC17/13 interactions in SUS between WT SNAP25b, SNAP23, CSPα, E-STOP, and CLIP3 and mutated Ψβ*XX*QA versions of these proteins. This analysis confirmed that all proteins tested interact with full-length zDHHC17/13 and that the conserved proline within their Ψβ*XX*QP motif is important for these interactions (Fig. 4*A*). Consequently, focusing on the interaction of HTT_1–550_ with zDHHC17/13 in SUS, we studied the effect of individual alanine substitutions within amino acids 494–502 of HTT. With this analysis, we found that although some amino acid substitutions outside the Ψβ*XX*QP consensus (*i.e.* T496A and R500A) can impair zDHHC17/13 interaction, I495A, Q498A, and P499A within the Ψβ*XX*QP motif have the most detrimental effect on zDHHC17/13 binding (Fig. 4*B*). Alanine substitutions of equivalent positions of SNAP25b (Val^113^, Gln^116^, and Pro^117^) also resulted in major loss of SNAP25b binding to zDHHC17/13 (Fig. 4*C*), consistent with previous effects of these mutations on membrane targeting and zDHHC17-mediated *S*-acylation of SNAP25 (37). A less prominent reduction of SNAP25b binding to zDHHC17/13 was also observed with V112A mutation, which could possibly explain the efficient zDHHC17/13 interaction of E-STOP, which has an alanine at equivalent position. Importantly, none of these four mutations in SNAP25 influenced its relatively weak binding to zDHHC3 (Fig. 4*C*), demonstrating that the Ψβ*XX*QP consensus is specifically required for interaction with AR domains of zDHHC17/13. Further amino acid substitutions of Pro^117^ in SNAP25b indicated that the hydrophobicity of this proline is required for AR binding, because valine or isoleucine substitutions partially restored zDHHC17/13 interaction, whereas glycine, serine, or threonine substitutions did not (Fig. 4*C*). These results also come in agreement with the effect of various Pro^117^ substitutions on membrane targeting of SNAP25b (37).

##### The GVVASQPARV Sequence of SNAP25b Specifically Recognizes the AR Domains of zDHHC17 and zDHHC13

**FIGURE 5.**
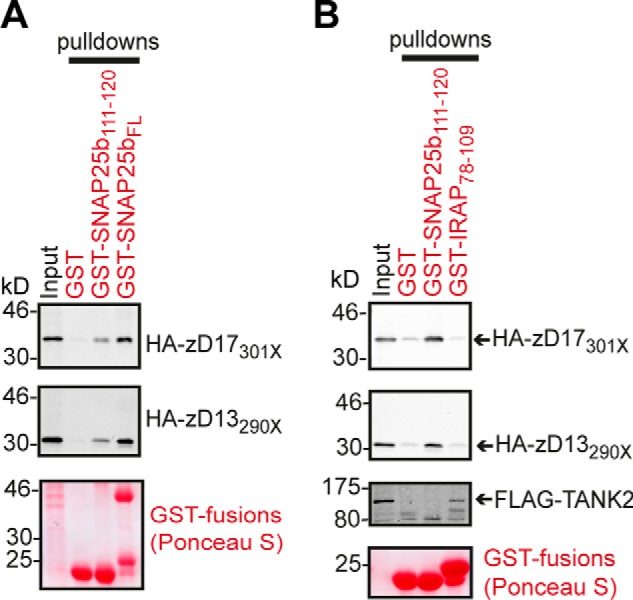
**A GVVASQPARV sequence of SNAP25b is sufficient for specific recognition by the AR domain of zDHHC17/13.** *A*, HA-tagged AR-containing fragments of zDHHC17 and zDHHC13 (STOP codons on cDNAs introduced at corresponding amino acid positions marked with *X*) were expressed in HEK293T cells, and their ability to interact with the GVVASQPARV peptide (SNAP25b_111–120_) and full-length SNAP25b (SNAP25b_FL_) was assessed by pulldowns of corresponding lysates by GST, GST-SNAP25b_111–120_, and GST-SNAP25b_FL_. One-twelfth of total inputs and one-quarter of total bound fractions were run on 12% gels, and following transfer, blots were stained by Ponceau S solution and probed with an HA antibody. *B*, HEK293T cells expressing HA-zD17_301X_, HA-zD13_290X_, or FLAG-TANK2 were lysed, and the binding preference of these AR proteins to a Ψβ*XX*QP (SNAP25b peptide) or a R*XX*DPG (IRAP peptide) sequence was assessed by pulldowns of corresponding lysates by GST, GST-SNAP25b_111–120_, and GST-IRAP_78–109_. Bound fractions and one-twelfth of total inputs were run on 12% gels, and following transfer, blots were stained by Ponceau S solution and probed with either HA or FLAG antibodies.

To assess whether a short peptide containing a Ψβ*XX*QP sequence is sufficient for recognition by the AR domains of zDHHC17/13, a 10-amino acid SNAP25b peptide (SNAP25b_111–120_) containing the Ψβ*XX*QP sequence (GVVASQPARV) was appended to the C terminus of GST and compared with full-length GST-tagged SNAP25b (GST-SNAP25b_FL_) for its ability to capture the AR domains of zDHHC17/13 from transfected HEK293T cell lysates (Fig. 5*A*). We found that both GST-SNAP25b_FL_ and GST-SNAP25b_111–120_ were able to pull down HA-tagged AR-containing fragments of zDHHC17/13 (HA-zD17_301X_ and HA-zD13_290X_), whereas virtually no binding to GST was detected by either zDHHC17 or zDHHC13 proteins (Fig. 5*A*). To examine the specificity of binding of this short sequence to the ARs of zDHHC17 and zDHHC13, we also assessed whether this peptide can bind to another AR protein, like TAnkyrase-2 (TANK2), as well as whether zDHHC17/13 ARs can interact with TANK2-binding sequences. For this reason, we used a GST-fused 32-amino acid sequence of IRAP (IRAP_78–109_) containing the R*XX*DPG-binding motif of TAnkyrase-1 and -2 (41) and assessed binding of GST, GST-SNAP25b_111–120_ and GST-IRAP_78–109_ to either HA-zD17_301X_, HA-zD13_290X_, or FLAG-TANK2 from transfected HEK293T cell lysates (Fig. 5*B*). Indeed, we found that zDHHC17/13 binding was highly specific for the GVVASQPARV sequence of SNAP25b, whereas TANK2 bound only to its cognate IRAP peptide (Fig. 5*B*).

##### Existence of Potential AR-binding ΨβXXQP Sequences in Other zDHHC17-interacting Proteins

To identify other proteins that are highly likely to interact with zDHHC17/13 via similar AR-binding linear sequences, we searched for the presence of unstructured Ψβ*XX*QP sequences in physiologically relevant zDHHC17/13 interactors, among the few dozen established and putative zDHHC17/13-binding proteins (9, 24, 35) and their related homologues (see “Experimental Procedures”). We found that apart from the 6 proteins tested, 14 other zDHHC17-binding proteins also have such motifs within regions predicted to be disordered (Table 1). We further divided this motif into three submotifs based upon our observation that: (*a*) there was an additional nonvariable aliphatic residue when Pro is at position Ψ, and (*b*) there is a Gln present immediately upstream of the motif when Thr is at position β: These submotifs are [VIA][VI]*XX*QP, P[VI][VIL]*X*QP, and Q[VI]T*XX*QP. Among the proteins found were the neuronal kinases JNK1–3, which have been previously reported to interact with both zDHHC17 and zDHHC13, and with the interaction of JNK3α2 mapped to the AR domain of zDHHC17 (9); the Golgi-targeted GTPase-activating protein for Cdc42 (ARHGAP21) (42), which although not shown to interact with zDHHC17, its *S*-acylation is significantly reduced in mice lacking zDHHC17 (34); the zDHHC17 substrate, NMNAT2 (Nicotinamide mononucleotide adenylyltransferase 2), which is required for axon survival (43, 44); six of seven members of the Sprouty domain signaling proteins SPRY1–4 and SPRED1–2 (45, 46); and the essential for lipid homeostasis, endoplasmic reticulum, and Golgi-localized, SREBP1 and -2 (sterol regulatory element-binding proteins 1 and 2) (47–49). Of these 20 zDHHC17-interacting proteins with unstructured Ψβ*XX*QP sequences, 12 have their motifs conserved among human, frog, and zebrafish species; 8 have been shown to interact with zDHHC13 as well; and 15 have been previously shown to be *S*-acylated, of which 10 are known to be zDHHC17 substrates (Table 1).

**TABLE 1:**
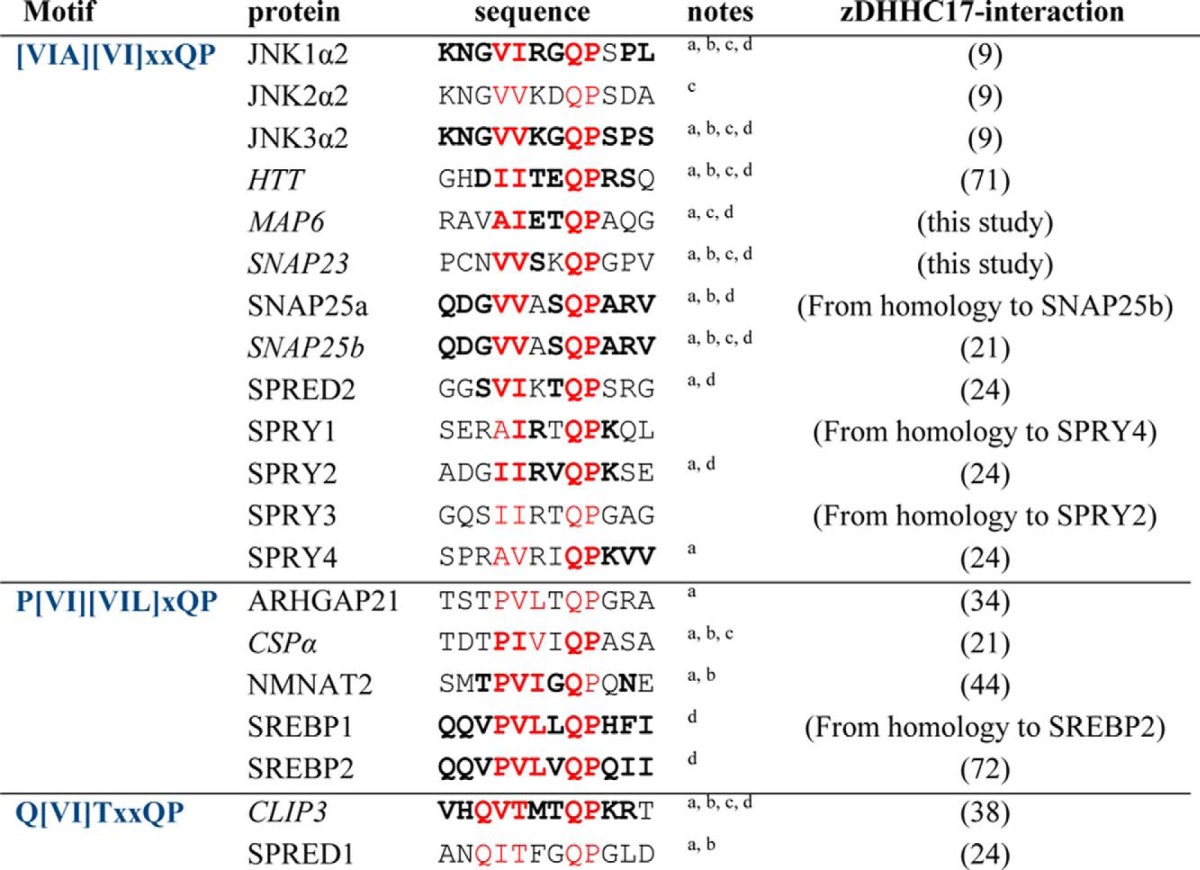
**Known and putative zDHHC17-interacting proteins having a [VIA][VI]*xx*QP, P [VI][VIL]*x*QP, or Q[VI]T*xx*QP motif within an unstructured region** Human sequences of these protiens containing the motif are shown. Protiens analysed in this study are shown in italics. Absolutely and highly (Val/Ile or Ser/Thr) conserved amino acids among human, frog, and zebrafish homologues are shown in bold type, where such sequences and homologues are present.

*^a^* Shown before to be palmitoylated.

*^b^* Established substrates of zDHHC17.

*^c^* Shown before to interact with zDHHC13 as well.

*^d^* Motif conserved among human, frog, and zebrafish homologues.

## Discussion

In this study we have identified variations of a novel unstructured peptide motif in many proteins, which is specifically required for binding to the AR domains of zDHHC17 and zDHHC13. This newly identified peptide motif seems to be a disordered interaction module falling into the group of a SLiM (short linear motif). SLiMs are short sequences (usually 3–10 amino-acids with 3–4 key residues involved in binding) that interact with structural features of other proteins; their short length means that: (*a*) they can bind their binding partners transiently and reversibly because of low affinity binding (with typical equilibrium dissociation coefficients in the range of 1–150 μm), and (*b*) they can exist in many unrelated proteins (50–52). AR recognition of SLiMs has recently emerged as a common feature shared by many AR-containing proteins, including: the chloroplast signal recognition particle protein (53), G9a/GLP methyltransferases (54), various homologues of ankyrin proteins R, B, and G (55, 56), TAnkyrase-1 and TAnkyrase-2 (57), and the ankyrin repeat family A proteins ANKRA1 and ANKRA2 (58). Moreover, a number of phylogenetically unrelated proteins have been found capable of binding to the same AR site of TAnkyrase or the same AR site of ankyrin repeat family A protein because of their related amino acid sequences in these unfolded regions (57, 58). Similarly, thephylogenetically unrelated SNAP25b, CSPα, HTT, CLIP3, and MAP6 can also bind to zDHHC17 and zDHHC13, because of the existence of similar zDHHC17/13 AR-binding sequences, within unstructured regions of these proteins. The intrinsic disorder of these sequences can be advantageous for interaction for a number of reasons: (*a*) the flexibility of the peptides allows complementary binding to target structures without steric restrictions, (*b*) a high rate of binding with low affinity and high specificity is achieved, and (*c*) a greater number of available sequences can be used for binding (59). As a result, the AR domain of zDHHC17/13 can engage in numerous interactions with a plethora of proteins and with high association/dissociation rates.

The three most critical residues for recognition by the AR of zDHHC17/13 appear to be a Val/Ile at position β, and Gln-Pro, as shown for HTT and SNAP25b (Fig. 4). In addition, a 10-amino acid peptide of SNAP25b (GVVASQPARV) was sufficient for recognition by zDHHC17/13 AR domains (Fig. 5*A*). This peptide, although adequate for interaction, did not bind as strongly as full-length SNAP25b. This might reflect nonoptimal presentation of the peptide when appended to the C terminus of GST or the requirement for longer peptide sequences for maximal binding. The interaction of this 10-amino acid peptide with zDHHC17/13 AR domains was specific, because this sequence displayed no binding to TANK2 (Fig. 5*B*).

**FIGURE 6.**
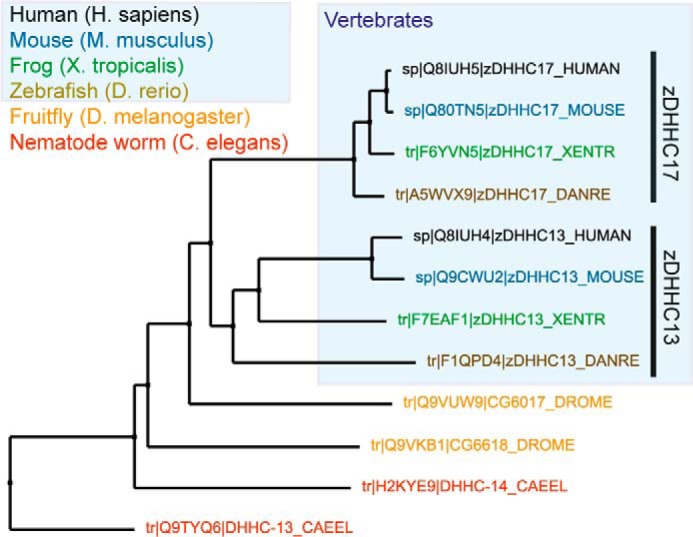
**Neighbor joining tree showing phylogenetic relationships of metazoan AR-containing zDHHCs.** Vertebrate zDHHC17 enzymes are more closely related to vertebrate zDHHC13 ones. UniProt IDs are shown. Protein sequences were aligned using CLUSTALW2, and tree was constructed using Jalview software.

The fact that vertebrate homologues of SNAP25b, SNAP23, CSPα, HTT, CLIP3, and MAP6 have high conservation within their Ψβ*XX*QP regions among distal vertebrate species suggests that these proteins have independently acquired these sequences for AR-zDHHC17/13 binding. Such a mechanism of convergent evolution has been thoroughly described for the appearance of ankyrin G-binding sequences in vertebrate KCNQ and Na_V_ channels (60), whereas other AR-binding sequences, like the ankyrin-binding motif of the L1 family of cell adhesion molecules (L1CAM), present in both mammals and nematode worms (61), appear to have emerged earlier in evolution. Among the six proteins tested in this study, SNAP25b and CSPα also have zDHHC17/13-binding [PV][VI]*XX*QP sequences conserved in some invertebrate species (V[VI]*XX*QP and P[VI]*XX*QP respectively), including*Drosophila* homologues (36, 62, 63); the existence of such sequences could explain the neuronal functions and *S*-acylation activity of the *Drosophila* zDHHC17 homologue, HIP14 (CG6017), toward these substrates (64, 65). A phylogenetic tree among established metazoan AR-containing zDHHCs indicates closer phylogenetic relationships between vertebrate zDHHC17s and vertebrate zDHHC13s, with the *Drosophila* CG6017 being more related to vertebrate zDHHC17/13 than other invertebrate zDHHC proteins (Fig. 6). Collectively, the above suggest that all vertebrate zDHHC17/13s, and possibly *Drosophila* CG6017, share the features for Ψβ*XX*QP-binding, conceivably because of conservation of this feature from a common ancestor protein. Similarly, the related TAnkyrase-1 and TAnkyrase-2 AR proteins can both recognize R*XX*DPG sequences of target proteins (41, 57), and the ANKRA1 and ANKRA2 paralogs both recognize a P*X*LP*X*[IL] sequence in a diverse set of binding proteins (58).

Although most (75%) of the Ψβ*XX*QP-containing zDHHC17-interacting proteins have been previously shown to be *S*-acylated, only two-thirds of them (and half of the total) are also known to be zDHHC17 substrates (Table 1). Some of these proteins that are not known to be substrates of zDHHC17are either not *S*-acylated at all (JNK2α2) or have been shown to be *S*-acylated (MAP6) by enzymes other than zDHHC17/13 (66, 67). Moreover, zDHHC13 is unable to *S*-acylate some zDHHC17 substrates, despite interacting strongly with them (21). The above indicate that although Ψβ*XX*QP binding is usually linked with *S*-acylation, the latter process is not necessary a consequence of AR binding. Hence, binding to AR domains of zDHHC17 and zDHHC13 must serve additional to substrate recruitment functions, and one of these function is JNK activation, caused by simultaneous recruitment of MKK7 and JNK by zDHHC17/13 (9). Additionally, evidence exists that (one or many molecules of) zDHHC17 can participate in oligomeric complexes with HTT and other proteins (19, 24) for functions that are currently unknown but seem to include enhancement of zDHHC17 *S*-acylation activity (19). Because zDHHC13 can recognize the same motif in HTT and other proteins, it is highly probable that similar complexes exist for zDHHC13 too. Furthermore, the loss of either zDHHC13 or zDHHC17 in mice results in similar Huntington-like neuropathological deficits (14, 15), despite zDHHC13 being less active than zDHHC17 (20, 38) or not active at all (18, 21, 44, 68) toward the vast majority of zDHHC17 substrates; therefore, it is very likely that many neuronal functions of these two zDHHC enzymes derive from molecular functions linked to AR binding, which are supplementary to, or independent of zDHHC17/13 *S*-acylation activity.

Many of the identified proteins with a Ψβ*XX*QP sequence contain serine(s) or threonine(s) within the variable amino acids of the sequence (Table 1). Because phosphorylation events seem to be enriched within intrinsically disordered regions of proteins (69, 70), it is plausible that some Ser/Thr residues in zDHHC17/13-binding proteins get phosphorylated. Negative regulation of AR binding by serine phosphorylation has been previously documented for HDAC4 binding to the AR domain of ANKRA2 (58). Similarly, phosphorylation of Ser/Thr residues may positively/negatively affect binding of proteins to the AR of zDHHC17/13. For instance, the loss of binding of HTT-T496A mutant (Fig. 4*B*) may be attributed to loss of Thr^496^ phosphorylation; alternatively, this or other Ser/Thr residues may be involved directly in AR binding. Extensive mutagenesis and trial of numerous peptides of different length is likely to uncover the contribution of nonconserved amino acids within Ψβ*XX*QP sequences on zDHHC17/13 AR binding. In-depth analysis of amino acid preferences within this newly identified AR-binding motif is likely to lead to the identification of additional zDHHC17/13-interacting proteins, new zDHHC17/13 substrates, novel functions deriving from *S*-acylation-independent binding events, and hence further elucidation of cellular and physiological roles of these two *S*-acyltransferases in the brain and other organs.

## Author Contributions

L. H. C. and K. L. designed the experiments. M. C. S.-P. produced the His_6_-tagged proteins used in this study. K. L. performed all the experiments. K. L. and L. H. C. wrote the manuscript. All authors approved the final version of the manuscript.

## Footnotes

2 The abbreviations used are:

zDHHC: zinc finger DHHC
AR: ankyrin repeat
NTA: nitrilotriacetic acid
SUS: split ubiquitin system
Ade: adenine sulfate
PLV: protein A-LexA-VP16.
